# Impact of COVID-19 on the wellbeing of micro and small entrepreneurs of rural Pakistan

**DOI:** 10.3389/fpubh.2022.993412

**Published:** 2022-10-13

**Authors:** Wajid Khan, R. M. Ammar Zahid, Ikram Ullah, Muhammad Asif Chuadhry, Saqib Yaqoob Malik, Yasir Hayat Mughal, Nazia Batool, Abida Begum, Heesup Han, Abdullah Mohamed

**Affiliations:** ^1^Department of Business Management, University of Baltistan, Skardu, Pakistan; ^2^School of Accounting, Yunnan Technology and Business University, Kunming, China; ^3^Department of Economics, University of Malakand, Chakdara, Pakistan; ^4^Department of Management Sciences, Shifa Tameer-e-Millat University, Islamabad, Pakistan; ^5^Department of Health Administration, College of Public Health and Health Informatics, Qassim University, Al-Bukayriyah, Saudi Arabia; ^6^School of Marxism, Northeast Forestry University, Harbin, China; ^7^College of Hospitality and Tourism Management, Sejong University, Seoul, South Korea; ^8^Abdullah Mohamed Research Centre, Future University in Egypt, New Cairo, Egypt

**Keywords:** COVID-19 pandemic, social wellbeing, micro and small businesses, household expenditures, Pakistan

## Abstract

According to the constitution of Pakistan, the state is responsible for the provision of necessities of life to its citizens whenever their livelihood is permanently or temporarily threatened. COVID-19 and its associated lockdowns were a series of events where amenities of life around the world were seriously endangered. Especially, hard hit were the small- and medium-sized entrepreneurs (SMEs) of rural Pakistan. To quantitatively assess the social and economic impact of COVID-19, we interviewed the local microenterprise owners in rural Pakistan from January to February 2021 and then June 2021. Mean comparison tests were estimated for pre- and post-COVID-19 periods. Results reveal that the COVID-19 pandemic has significantly and negatively affected wellbeing of micro and small entrepreneurs in the regions as the income of most of the sampled entrepreneurs significantly decreased during the pandemic. Disaggregated consumption analysis however revealed that nominal consumption of food, clothing, energy, health, and education all increased, except for communication, during the pandemic. Furthermore, the regression analysis revealed that changes in income, occupation, borrowing during COVID-19, and family type of the respondents were significant factors in mitigating the effects of COVID-19. Based on the findings, policy recommendations are also spelled out in the last section.

## Introduction and background

In December 2019, the first case of COVID-19 was reported in Wuhan, China. The first case outside China was reported on January 13 in Thailand (1), and by the end of January 2020, the total number of COVID-19 infected cases reached an overwhelming 7,819 people spread throughout the entire world (2, 3). According to the latest statistics available (May 18, 2022), the total number of cases in Pakistan stood at more than 1.5 million people, causing a total of more than 30,000 deaths (4). To slow down the spread of the pandemic, Pakistan imposed lockdowns in March 2020, which were subsequently eased in May 2020.

Besides health, COVID-19^1^ has severely affected businesses around the globe, especially in developing countries (5, 6). During COVID-19, the global Gross Domestic Product (GDP henceforth) declined for the first time in more than 25 years (7). The GDP of India declined the most (23.9%), followed by the Eurozone (12.1%), Brazil (9.7%), United States (9.5%), and Japan (7.8%). During the same period, the growth in Pakistan's GDP declined to 1.4% (7). The economic downturn during this period pushed many people around the world below the poverty line. For instance, a simulation analysis by Sumner et al. (8), covering 138 low-income and 26 high-income countries, estimated that COVID-19 could drive another 85 million into poverty (8, 9). These predictions are empirically proved correct by many studies from around the world [see for instance (10, 11)].

The negative economic impacts of COVID-19, although generally severe, were not equally distributed across sectors and job types. The likelihood of losing their jobs was the greatest for non-permanent employees (5) and the likelihood of slipping below the poverty line was the greatest for self-employed people (10). Likewise, sectors connected with international trade and tourism (such as accommodation, food services, and transportation) suffered the most as compared to agriculture and construction (10). Moreover, as reported by Sonobe et al. (5), the negative impacts of COVID-19 on countries also varied with their level of national income. Countries dependent on micro, small, and medium enterprises (MSMEs henceforth)^2^ for the bulk of employment generation and having a lower level of national income are likely to be the hard hit in terms of negative economic consequences of COVID-19. MSMEs usually have less control over resources and are more exposed to shocks (13, 14).

Early research shows that the lockdowns associated with COVID-19 harmed 58% of the medium-sized, 65% of the small, and 69% of the micro-sized firms (15). Likewise, Pedauga et al. (16) reported that MSMEs explained 62% of the employment reduction in Spain due to COVID-19-related lockdowns. The negative effects are however not only transitory but may lead to bankruptcy (17) and market exits (18) for some of the business firms. MSMEs are the leading employment provider sector in Pakistan, which represent an estimated 90% of all business and contribute 40% to GDP and the country's export (19). Statistics show that COVID-19 has significantly affected the economic activity in Pakistan (7, 20) but less is known about how the negative consequences of COVID-19 on MSMEs translated into reduced welfare at the rural level^3^.

Foregoing in view, the current study is carried out to understand the negative economic impact of COVID-19 on small entrepreneurs in rural Pakistan. To the best of our knowledge, this is the first attempt to study the impact of COVID-19 on micro and small entrepreneurs' wellbeing in this region. The rest of the paper is organized as follows: Section Relevant literature, theoretical framework, and hypothesis development discusses the relevant literature, develops a theoretical framework, and deduces a testable hypothesis. Section Research design of the study outlines the research design, data, and analytical methodology of the study. Section Results and discussions presents results and analysis, while Section Conclusion and policy implementations concludes the study and presents policy recommendations.

## Relevant literature, theoretical framework, and hypothesis development

With the onset of COVID-19 and its associated lockdowns, the logistics and supply chain management disruptions were the foremost to be experienced by MSMEs (15). About half of the MSMEs experienced delays in the delivery of services and products and an unprecedented demand deficiency [see for example (15, 21)]. Since lack of demand affects MSMEs the most as compared to large firms (16), the majority of the MSMEs owners reported lack of customers as the most negative economic outcome of the COVID-19-related lockdowns (11, 22).

Deficient aggregate demand resulted in no/less sales and revenues for the MSMEs. For instance, Shinozaki and Rao (21) reported education- and construction-related firms with no sales during March 2020 in the Philippines to be 47.9% and 39.8% higher as compared to agriculture-based firms. Consequently, revenues of the MSMEs also followed the same path [see (18, 23)]. On the other hand, supply disruptions caused the cost of production for MSMEs to increase (22), resulting in reduced profits somewhere (22, 24) and in financial and accounting losses elsewhere (17, 25).

To minimize losses, the MSMEs natural response was to either completely shut down (18, 22) or to downsize production (16) by laying-off workers. Since employment predominantly depends on MSMEs (16), downsizing resulted in massive unemployment around the world. Other loss-minimizing strategies adopted by MSMEs during COVID-19 included forced unpaid leaves and reduced wages (14, 21, 25–27). Consequently, incomes of both the employer (due to demand deficiency and cost-push-induced reduced profits) and employees (due to unemployment, forced unpaid leave, or reduced wages) decreased, resulting in increased poverty (10, 28). The net effects of all such developments during COVID-19 resulted in reduced livelihood expenditure (22) and food insecurity (29, 30).

**Figure 1:**
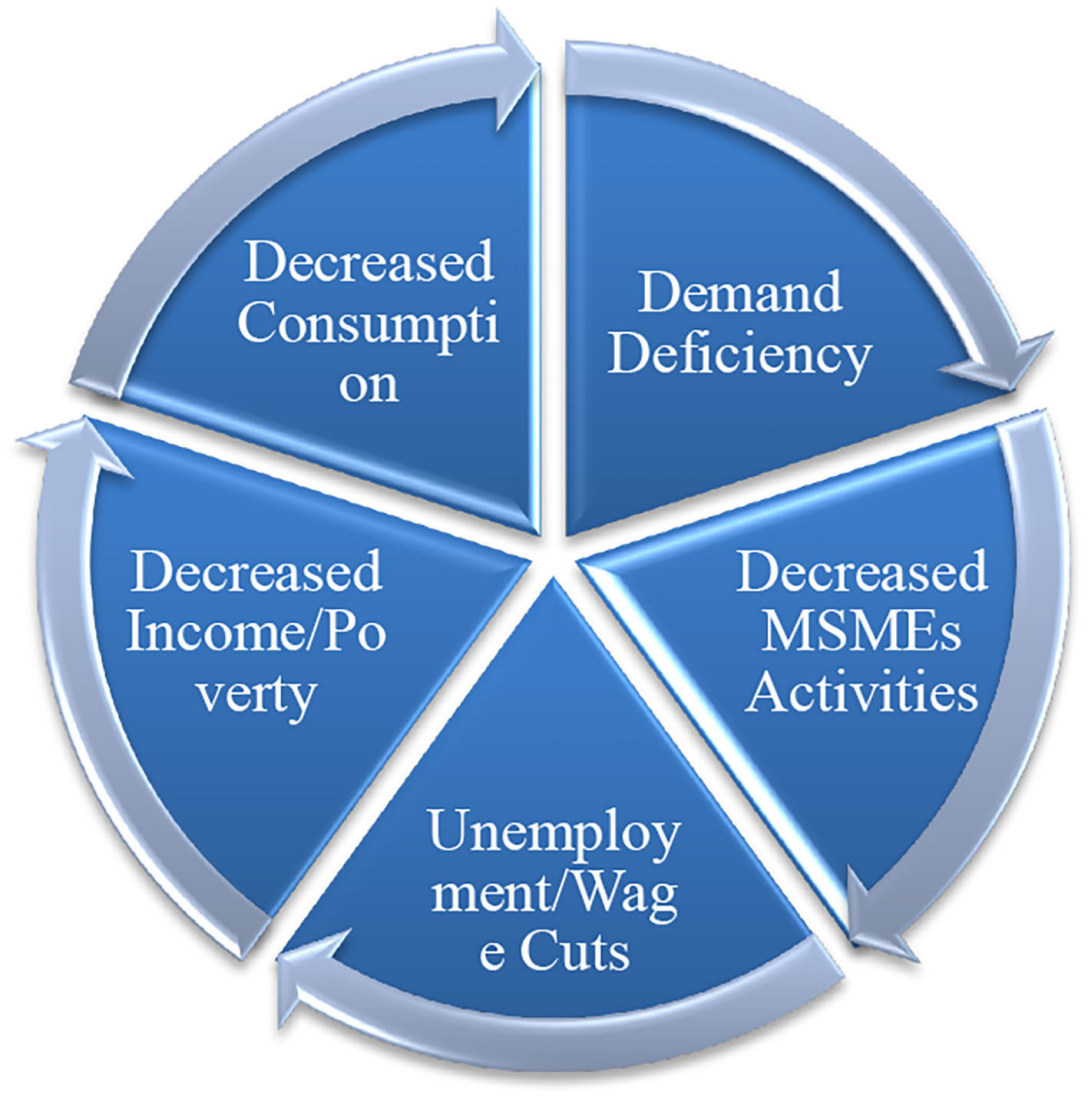
Theoretical framework of the study.

The theoretical framework, based on the early findings of the COVID-19 era empirical research and Keynesian economics, is detailed in Figure 1. This enables us to deduce the testable hypothesis that COVID-19 has a significant negative impact on the income of the economic agents, and in the absence of any intervention, the reduced income must translate into reduced household expenditure and welfare^4^.

## Research design of the study

### Area profile and sampling

District Dir (Lower) has 1.436 million people (Cense report 2017). The district has six tehsils that have 34 union councils. Out of the total population of the district, 0.31 million are active laborers. Agriculture accommodates about 40% of the labor force (31), while only 12% are self-employed. About 40% of the district labor force are wage earners.

Human capital with good education and sound health contribute substantially to the development of a society. Statistics show that Pakistan's health sector is lagging far behind the rest of the world. According to Pakistan's demographic health survey report (2012–2013), the child mortality rate is 74 out of 1,000; malnutrition in children under age 5 is 24% and only 34% of the women attend health facilities during birth. The situation in Khyber Pakhtunkhwa is even worse. According to PSLM (32), about 78% of children need treatment for diarrhea and 14% of women do not visit any medical facility during pregnancy. COVID-19 further increased stress on the already weakened health system of Pakistan and especially on the health facilities in rural areas (33).

### Sampling and data collection

Data regarding the number of small and medium enterprises in the province is not objectively known. According to some estimates [see for example (34)], the number of troubled small and medium enterprises is approximately 2,504 in the province, still, the overall number is not known. The district where the study is conducted is a tax-free zone and there is no registry of the small and medium enterprises in the district that could be used as a sampling frame. Consequently, the study uses a convenient and purposive sampling framework. Overall, data has been collected from 303 entrepreneurs engaged in business related to agriculture, forestry, mining, services, manufacturing, construction, storage, transport and communication, real estate and rental, and retail business. Respondents belong to three tehsils and 18 union councils, as shown in Table 1. A structured questionnaire with close-ended questions, adapted from Khan et al. (35), is used for data collection (Appendix).

**Table 1:** Union council-wise distribution of the sample units.

**Union** **councils**	**Sampling units**	**Tehsil**	**Sampling units**	**District**	**Sampling units**
Ouch	22	Adenzai	172	Dir lower	303
Kotigram	23				
Khanpur	12				
Tazagram	61				
Asbanr	8				
Chakdara	32				
Badwan	6				
Khadagzai	8				
Temergara Urban	14	Temergra	63		
Khungai	8				
Bandagai	9				
Bagh	11				
Talash	12				
Noorakhel	9				
Koto	8	Balambat	68		
Lajbook	22				
Munjai	17				

### Analytical framework

The data is analyzed using frequencies, descriptive statistics, and independent sample *t*-test for before–during comparisons to assess the welfare consequences of COVID-19. Subsequently, various consumption heads are used as dependent variables to know how variations in income, occupations, family type, business age, working hours, and borrowing during COVID-19 mitigated the welfare consequences of COVID-19. More formally, let *CFE*_*it*_ be changes in food expenditure of the *i*th MSME owner at the *t*th time (*t* = either *B* or *D*, where *B* refers to time before COVID-19 and *D* refers to time during COVID-19), *CII*_*it*_ be changes in income, *BDC*_*i*_ be borrowing during COVID-19 (= 1 if the owner borrowed money during COVID-19 and 0 otherwise), *ABY*_*i*_ be the age of the business at the time of data collection, *CIO*_*it*_ be changes in occupation during COVID-19, *CWH*_*it*_ be changes in working hours, and *FT*_*it*_ be the family type (= 0 if joint family and 1 if nuclear family) of the *i*th MSME owner. Then a regression model can be specified as;


(1) $$\begin{array}{l} CFE_{it}=\beta_{0}+\beta_{1}CII_{it}+\beta_{2}CIO_{it}+\beta_{3}CWH_{it}+\beta_{4}BDC_{i}\\ \;+\beta_{5}ABY_{i}+\beta_{6}FT_{i}+U_{it} \end{array}$$

Dependent on the error structure specified in equation (1)^5^, the study estimates a set of six such equations. Multicollinearity will be checked using the correlations matrix while heteroskedasticity and autocorrelation, respectively, will be checked using the White's (36) and Durbin-Watson tests.

## Results and discussion

Frequencies obtained from the data show that about 81 of our sampled respondents live in a joint family while the rest (19%) lives in nuclear families. Approximately, 90% of the respondents did not switch their jobs/businesses during the study period but 58% of them resorted to debt financing for running their activities and meeting household obligations. Interestingly, those who looked for alternative jobs/income sources were predominantly in the services (barbers and tailors) and agriculture sectors. As is shown in Table 2 (also see Table 3), however, the majority of the sampled respondents experienced a drop in their income level due to COVID-19.

**Table 2:** Income distribution before and during COVID-19 (Unit: Pakistani Rupee).

	**Before COVID-19**		**During COVID-19**	
	**Frequency**	**Percentage**	**Frequency**	**Percentage**
20,000 or less	1	0.33	18	5.94
21,000–25,000	10	3.3	27	8.91
26,000–30,000	32	10.56	28	9.24
31,000–40,000	99	32.67	111	36.63
41,000–55,000	100	33	57	18.81
56,000–70,000	24	7.94	48	15.84
71,000–85,000	31	10.23	10	3.30
86,000 or above	6	1.98	4	1.32

**Table 3:** Mean differences before and during COVID-19.

**Variable(s)**	**Mean (before COVID-19)**	**Mean (during COVID-19)**	**Mean Difference**	**Levene's Test^a^**	***t*-test**
Age of business	NA	6.6	NA	NA	NA
Working hours	8.65	8.17	−0.488	2.804	3.579*
Income	46,641.91	42,481.85	−4,160.07	0.178	3.258*
Expenditure on food	16,940.59	20,488.45	3,547.86	31.11*	−5.129*
Expenditure on clothing	2,894.06	3,616.5	722.44	5.82**	−3.34*
Expenditure on energy	5,990.43	6,956.11	965.68	4.04**	−1.91**
Expenditure on health	2,500	2,714.85	214.85	2.28	−1.18
Expenditure on communication	993.73	984.82	−8.91	2.80	0.20
Expenditure on education	2,601.32	3,441.91	840.59	4.86**	−5.67*

* p < 0.01,

** p < 0.05.

a The Levene's statistic tests the hypothesis that the two samples (before and during COVID samples) have equal variance. The t-test value then depends on whether the null hypothesis of equal variance is rejected or accepted. The last column of the table hence reports the relevant t-statistic.

The theoretical framework developed in Section Research design of the study implies that the negative economic consequences start with supply shocks and demand deficiency. The results outlined in Table 3 show that, on average, the working hours of the MSMEs have reduced from the pre-COVID era of 8.65–8.17 h, showing the slowing down of the rural economy of the area under consideration. Resultantly, incomes of the sample respondents also decreased but contrary to the postulation of the theoretical model, expenditure on most heads (except communication) showed an increasing trend. That is, Levene's and independent sample t-tests confirm that working hours and income significantly reduced during COVID-19, but expenditure on food, clothing, energy, and education significantly increased during the same period. Expenditure on health and communication, although changed, is, however, statistically insignificant during the study period. These apparently divergent results are further discussed once the influence of the mitigating factors on variations in consumption (Table 4) during COVID-19 is considered.

**Table 4:** Mitigating factors of consumption during COVID-19.

**Mitigating factors**		**Variations in consumption on**					
	**VIF**	**Food**	**Clothing**	**Energy**	**Health**	**Communication**	**Education**
Change in income	1.06	0.067*	−0.002	−0.043**	−0.02	0.003**	0.006
Change in working hours	1.03	133.48	56.74	92.03	−110.69	−47.25*	80.47
Change in Occupation	1.02	1.33	1,186.4*	849.72	1,385.1*	−277.26*	−86.18
Borrowing during COVID-19	1.11	531.23	598.61*	−1,337.4*	−514.5**	35.55	−189.4
Age of business	1.14	−30.07	43.26	−172.51*	3.97	1.002	−12.14
Family type	1.03	−2,240.8*	−124.42	−666.55	173.97	8.49	−626.97*
Intercept		5,992.8*	−374.11	4,584.8*	455.45	−58.52	2,011.51*
R^2^		0.054	0.054	0.068	0.062	0.16	0.032
F-statistics		2.78*	2.86*	3.60*	3.29*	9.19*	1.6
Durbin-Watson d-statistics		1.85	1.92	2.15	1.99	1.5	1.74
White's statistics^a^		11.71**	5.89	5.46	6.45	25.95*	7.46

* p < 0.01,

** p < 0.05.

a The null hypothesis of the White's general heteroskedasticity is that the errors are homoscedastic. In case of rejection of the null hypothesis, heteroskedasticity and autocorrelation robust standard errors, and the corresponding t-ratios, are estimated.

The estimated regression results, based on variants of equation (1), are outlined in Table 4. Note that the correlation matrix of the mitigating/explanatory variables is not reported but none of the bivariate correlation coefficients exceeded 0.3 in absolute terms. Besides, the variance inflating factors (centered) are reported in Table 4. Hence, there is no issue of multicollinearity in the regression. Moreover, the *R*^2^ and the *F*-test associated with each regression implies that the inclusion of the mitigating factors significantly improves the fit of the model in comparison to an intercept-only model^6^. Although *R*^2^ associated with these models are relatively low, these are normally in the acceptable range (37).

The Durbin-Watson d-statistics values are in the acceptable margin (except when variations in consumption on communication is used as a dependent variable), implying that there is no issue of serial/autocorrelation. Likewise, heteroskedasticity is found only in the food consumption and communication regressions. The two models are therefore estimated using the heteroskedasticity and autocorrelation (HAC) robust standard errors.

The results outlined in Table 4 are generally in line with prior research. To save space, we only discussed the significant results outlined in Table 4. As postulated in the theoretical model, changes in income are positively related to variations in food consumption. Changes in working hours, occupation, and borrowing, although positively influencing food consumption, are all insignificant statistically. Family type (living in a nuclear family), however, influences food consumption negatively. This result is in line with Galeana et al. (24) and Dagpin et al. (18), to whom family works as a shock absorber during negative shocks like COVID-19. Since people living in joint families have shared responsibilities, as well as their sources of income may also be diverse, hence entrepreneurs living in nuclear families are at a disadvantage in such times.

Consumption of clothing is however influenced only by changes in occupation and borrowing during COVID-19. Although there were very limited opportunities for alternative income sources during COVID-19, prior research has reported that approximately 9–32% of business owners in South Africa were able to generate alternative incomes during COVID-19 (25)^7^. As mentioned in the starting paragraph of this section, those who looked for alternative income sources were predominantly from the services sector (barbers and tailors) whose businesses were strongly dependent on interactions with others and could have caused spreading the disease to others. Hence, their businesses were more affected, and they looked for alternative jobs. Energy consumption (firewood, electricity, fuel, and gas) was however negatively influenced by changes in income, age of the business, and borrowing during COVID-19. This may be because food consumption was essential for survival and may have crowded out other consumption during the period. That is, whatever slight increase in income or borrowing occurred during that period was solely devoted to food consumption.

Variations in expenditure on health before and during COVID-19 were positively influenced by a change in occupation but negatively by borrowing during COVID-19. Variations in expenditure on communication, on the other hand, were positively influenced by income and negatively by working hours and change in occupation. While the impact of income and occupation on expenditure on communication is standard, the negative impact of increased working hours on communication expenditure is paradoxical. Last, although the fit statistics warrant a poor fit, variations in education expenditure before and during COVID-19, according to the results, depended only on the family type. Again, MSMEs owners living in nuclear families reduced their expenditure on education, as compared to those living in joint families, during the COVID-19 era.

Before closing this section, the results outlined in Table 3 showed that working hours and incomes of the MSME owners significantly decreased during COVID-19 but consumption of food, clothing, energy, and education increased during the same period. Although it is logical in the circumstances that some of the expenditure may have increased^8^, these apparently counterintuitive results may be because the study considers nominal values of the variables under consideration. According to Planning Commission of Pakistan reports (May and July 2021), prices of food items and energy increased by double digits during the period under study. Accounting for this much inflation would further decrease the real value of income. An increase in real consumption may also be negative during the same period if inflation is taken care of. Indeed, prior research has also shown that supply-related disruptions resulted in cost escalations around the world [see (15, 22)]. The net economic effects of COVID-19 may even be more severe on the welfare of the MSME owners if cost escalations are made part of the calculus.

The other counterintuitive result relates to the impact of borrowing on various expenditure heads (38). Specifically, the results show that borrowing during COVID-19 increased expenditure on clothing but those on energy and health were decreased. Since it has been already stated that none of the MSMEs in the area are registered because the area under study is a tax-free zone. But being unregistered also implies that these MSMEs cannot resort to formal credit sources for financing their needs. Consequently, informal sources of credit are tapped in times of emergencies. Hence, it is quite possible that during the study period, the respondent may have experienced no health emergency. The credit taken by the respondents might have been used elsewhere, such as a cultural, social, or religious obligation. It is also quite possible that credit from one informal source during COVID-19 is obtained to pay back another informal source from whom credit in an earlier time is obtained.

## Conclusion and policy implementations

Prior research from around the globe has shown that COVID-19 and its associated lockdowns have devastating economic effects on the masses. Especially hard hit, according to the research, are the MSMEs and areas that are less economically privileged. To assess the economic impact of COVID-19 on the owners of MSMEs in rural Pakistan, we have collected data from 303 owners spread over three tehsils of district Dir Lower of Khyber Pakhtunkhwa, Pakistan. The data were then analyzed using descriptive statistics and rigorous regression analysis.

The results show that, as in other geographical regions of the world, working hours and nominal incomes of the MSME owners were reduced significantly by the onset of COVID-19 and its associated restrictions. Paradoxically, it was found that nominal expenditure on food, clothing, energy, health, and education increased and those on communication decreased during COVID-19. Given that inflation in Pakistan was in double digits, it may be the case that the real value of income and consumption and the associated wellbeing may be lower than estimated. But if these values are considered as they are, then in line with previous research [see (21, 25)], the MSME owners may have dipped their savings or retained profits to smoothen their consumptions. Again, this interpretation is welfare reducing as such developments will harm their businesses in the longer run.

Moreover, this study also found that variations in income caused positive changes in the consumption of various food items. Likewise, variations in working hours have also positive effects, although insignificant, on most of the consumption items. Informal borrowing during COVID-19 increased consumption of clothing, food (insignificantly), and communication but reduced consumption of energy and health, this may be due to the nature of the “informal” borrowing. These findings have important policy implications for coping with the negative economic consequences of emergencies like COVID-19.

Since the theoretical framework and the findings of the study are very much in line with Keynesian Economics, the policy implications also are of the Keynesian type. And indeed, many countries like the USA, China, and Pakistan followed such policies during COVID-19. Loans are waived off to support micro-, small-, and medium-sized enterprises considerably. But, given that the MSMEs in the area under study have no access to formal credit during the study period, it is recommended that the MSMEs should be brought under the formal credit nets to help them in times of emergencies. Moreover, MSMEs around the world benefited from online sales and digitalization during COVID-19. To continuously reap the employment generation benefits of the MSMEs in Pakistan, the owner must be trained in digitalization and online sales of their products.

Before closing the study, there are several limitations of the study that needs to be mentioned. First, the sample consists of micro and small entrepreneurs working in lower Dir Khyber Pakhtunkhwa, Pakistan. Future studies may include entrepreneurs from other districts and provinces to accurately understand the negative impact of COVID-19 on micro and small businesses. Second, the assessment in the instant study is solely based on quantifiable parameters. Future research can add psychological, environmental, and social impacts to get a holistic view of the COVID-19 impacts. Third and most importantly, the study does not capture the gender aspect of COVID-19's impacts on SMEs. Recently, it has been that women-led MSMEs faced more serious impacts than men-led MSMEs (21) and that such MSMEs were less likely to receive public support (39). MSME's ownership is predominantly in the hands of men in the study area, but future research must not ignore this gender aspect of the impacts of COVID-19.

Fourth, the study relied on the memory of the respondents for information. Since the two time periods were 6 months apart, there may be an element of memory decay in the information provided. Likewise, given the expectations of MSME owners in times of emergency, it is quite possible that the owner might have understated their incomes and overstated their consumption. Future researchers are advised to adopt novel data collection techniques to overcome such limitations.

## Data availability statement

The raw data supporting the conclusions of this article will be made available by the authors, without undue reservation.

## Author contributions

RZ, WK, and NB contributed to conception and design of the study. SM and AB organized the database. RZ and IU performed the statistical analysis. WK wrote the first draft of the manuscript. YM, HH, MC, and AM wrote literature sections of the manuscript. All authors contributed to manuscript revision, read, and approved the submitted version.

## Funding

This project was partially financed by the University of Baltistan, Skardu, project number UOBS-ORIC/Covid-2020/004.

## Conflict of interest

The authors declare that the research was conducted in the absence of any commercial or financial relationships that could be construed as a potential conflict of interest.

## Publisher's note

All claims expressed in this article are solely those of the authors and do not necessarily represent those of their affiliated organizations, or those of the publisher, the editors and the reviewers. Any product that may be evaluated in this article, or claim that may be made by its manufacturer, is not guaranteed or endorsed by the publisher.

## Footnotes

^1^Covid-19 is the disease caused by the virus known as SARS-CoV-2. SARS-CoV-2 spread was reported in late 2019 in Wuhan, China, and has since spread all over the world.

^2^Micro and Small, and medium enterprises (MSE): According to SBP (12), micro-enterprises are those firms that have less than 10 employees (excluding seasonal labor), while small enterprises are those having up to 50 employees, including contractual employees and an annual turnover of PKR 150 million. Medium-sized enterprises are categorized business-wise; first, manufacturing and service have employees (51–250) and annual sales turnover of over PKR 150–800 million. Second, trading has 51–100 employees and an annual sales turnover of over PKR 150–800 million.

^3^Approximately, 70% of Pakistan's population still lives in rural areas.

^4^According to Friedman's permanent income hypothesis (1957), transitory income changes should not change consumption and welfare. But given the uncertainties associated with the Covid-19 outbreak, changes in income during that period could not be considered transitory changes.

^5^If the error structure satisfies the usual assumptions of classical linear regression model, the equation can be estimated using the Ordinary Least Squares method and the resulting estimates are BLUE.

^6^When treating changes in education as a dependent variable (the only case in the six regressions), the F-value is insignificant, implying that the model provides no improvement in comparison to an intercept only model.

^7^The alternatives included selling masks, food items and helping elderly and those in quarantine due to Covid-19.

^8^This may be true as most of the family members remained in homes during lockdowns, which might have increased demand for food and energy.
