# Supplementary material for: Impact of COVID-19 on the wellbeing of micro and small entrepreneurs of rural Pakistan
Source: Front Public Health. 2022 Oct 13;10:993412. doi: 10.3389/fpubh.2022.993412 (PMC9609786; doi:10.3389/fpubh.2022.993412)
Supplement: Supplementary file 1 [file Data_Sheet_1.docx]

**Appendix: Household Survey for assessing the Entrepreneurial activities and Covid-19: Empirical Evidence from Dir Lower.**

**Notes: The questionnaire contains for those respondents who are running their own businesses (entrepreneurs)**

**A: Information about Borrowers**

| A01: **Borrowing/no Borrowing.**.............................................................................................................. | | |
| --- | --- | --- |
| A02: **Life of The Business**: ...................................................................................................................... | | |
| A03: **Occupation** | Before Covid-19 | During Covid-19/Current* |
|  |  |  |
| A04: **Working hours per day** |  |  |
| A05: **Income** |  |  |
| A06: **Family Type** | □Joint □Nuclear | □Joint □Nuclear |
| **Coding Scheme for A03:** 1. Agriculture and hunting, 2. Forestry and logging, 3. Mining and quarrying, 4. Services sector, 5. Wood and wood products, 6. The manufacturing industry, 7. Weapon industry, 8. Construction, 9. Transport, storage, and communication, 10. Real estate and rental, 11. Retail Business, 12. Business (trade), 13. Other personal services (barber, Tailor, etc), 14. Any other (Please specify)  **Coding Scheme for A06:** 1. Joint, 2. Nuclear | | |

B: Expenditures (Average Household Expenditure during last Year)

| Sr | Item | Average Before Covid-19 (Rs.) | Average During Covid-19 (Rs.) |
| --- | --- | --- | --- |
| 1 | Food expenditure |  |  |
| 2 | Clothes |  |  |
| 3 | Fuel |  |  |
| 4 | Firewood |  |  |
| 5 | Gas |  |  |
| 6 | Electricity |  |  |
| 7 | Health Expenses |  |  |
| 8 | Telephone/Mobile |  |  |
| 9 | Education |  |  |
